# The promotion of sleep wellness: Resilience as a protective factor

**DOI:** 10.3389/frsle.2023.1133347

**Published:** 2023-04-11

**Authors:** Alexa C. Allan, Alyssa A. Gamaldo, Charlene E. Gamaldo, Brian C. Gunia, Iyiad Mohamed Al Abdul Razzak, Edoghogho Ighodaro, Rachel Marie E. Salas

**Affiliations:** 1 Human Development and Family Studies, The Pennsylvania State University, University Park, PA, United States; 2 Department of Neurology, Johns Hopkins University, School of Medicine, Baltimore, MD, United States; 3 Johns Hopkins Carey Business School, Baltimore, MD, United States

**Keywords:** resilience, sleep disturbance, mental health, physical health, wellness

## Abstract

**Objectives:**

To evaluate the association between resilience, sleep quality, and health.

**Methods:**

This cross-sectional study included 190 patients (Mean age = 51, *SD* = 15.57) recruited from the Johns Hopkins Center for Sleep and Wellness. Patients completed a modified version of the brief resilience scale (BRS) to assess characteristics of resilience and questions to assess aspects of mental health, physical health, sleep quality, and daytime functioning.

**Results:**

Participants’ average score on the BRS was 4.67 (*SD* = 1.32, range = 1.17–7), reflecting a high level of resilience. There was a significant gender difference in resilience levels for men (Mean = 5.04, SD = 1.14) and women (Mean = 4.30, SD = 1.38), such that men reported significantly higher levels of resilience compared to women (*t* (188) = 4.02, *p* < 0.001) [lower levels of resilience were significantly associated with higher levels of (current) fatigue and tiredness after adjusting for demographic, physical, and mental covariates. In those reporting between one and three mental health symptoms, high levels of resilience minimized the negative influence that these symptoms had on sleep quality. This minimizing effect was no longer evident in those experiencing >3 mental health symptoms, who also reported significantly higher symptoms of fatigue despite their high resilience scores.

**Conclusions:**

This study emphasizes how resilience may affect the relationship between mental health and sleep quality in sleep patients. Resilience may further our understanding of the inter-relationships between sleep and the manifestation of physical health symptoms, a relationship that will likely heighten in relevance during personal and global crisis. An awareness of this interaction could be used as a proactive prevention and treatment strategy. In other words, incorporating methods to evaluate resilience in patients with mental illnesses regularly can be useful for predicting the potential manifestation and severity of sleep disturbance. Therefore, strategies that focus on promoting resilience could improve health and wellness.

## Introduction

Sleep plays a critical role in social, mental, and physical functioning. Overall functioning has been measured recently with constructs such as resilience (Buysse, 2014), which can be defined as the individual’s ability to successfully adapt in life despite social disadvantage or other highly adverse conditions (Pecillo, 2016). Resilience can be viewed as a biopsychosocial construct that is influenced by the interaction of biological (i.e., growth factors and genes), psychosocial (i.e., stress and depression) and environmental factors (i.e., economic burden and emotional support) (Karol, 2002; Davydov et al., 2010). Sleep may help or hinder resilience. For example, high-quality sleep can enhance neurobiological functions such as cognition and emotional stability (Ellenbogen, 2005; Haack and Mullington, 2005). Sleep disturbance has also been implicated in the manifestation of biological and psychosocially influenced medical conditions such as obesity, diabetes and stroke (Cappuccio et al., 2010; Benjamin, 2020; Malhotra, 2020) as well as conditions with strong interactive biological, environmental, and psychological influences such as depression, anxiety, and aggressive and violent behavioral patterns (Pallesen et al., 2010; Clinkinbeard et al., 2011; Chattu et al., 2019).

The manifestation, maintenance, and mitigators of resilience has taken on greater importance and increased attention in the face of the global COVID-19 pandemic crisis that has infected, at the time of this manuscript preparation, ~100 million people in the U.S. and 654 million people worldwide and resulted in the loss of ~1.1 million Americans and 6.7 million citizens globally (Dong et al., 2020). Disrupted sleep, which broadly characterizes the symptoms associated with insomnia, insufficient sleep, or poor sleep quality, has been recognized as a contemporary challenge around the world (Steptoe et al., 2007). In fact, more than a million American adults are affected by sleep disorders, while 17 and 40 million U.S. adults experience depressive episodes and anxiety, respectively (Evernorth Health Inc, 2022). Prescription patterns provide further evidence for the relevance of and associations among resilience, sleep, and affective stability. For example, around the beginning of the COVID-19 pandemic in the U.S, a 15% spike in sleep aid prescriptions constituted more than three quarters of all new antidepressant, antianxiety, and anti-insomnia prescriptions filled (Evernorth Health Inc, 2022). Evidence linking poor sleep to reduced resilience can be found in previous studies of “poor sleepers,” who are more likely to experience negative emotions for a longer period of time (i.e., less resilience) following an acute life stressor, as compared to “good sleepers,” who are more likely to report emotions demonstrating better adaptability and resilience (Steptoe et al., 2007). Thus, a relationship may exist between patterns of sleep and resilience in which either may serve to moderate or minimize the positive and negative impacts of the other. Furthering our understanding of the relationship can be of particular utility in combatting the short and potentially long-term impact of a global crisis (Evanoff et al., 2020; Gibson et al., 2020). This study aimed to: (1) evaluate the relationship between resilience and sleep, and (2) examine whether resilience may moderate the relationship between mental health symptoms and sleep.

## Methods

### Participants

This cross-sectional study included patients recruited from the Johns Hopkins Center for Sleep and Wellness. Each patient who signed a form indicating interest in research studies was approached by a study team member and consented using an Oral Consent Script approved by the Johns Hopkins Institutional Review Board (IRB 00108995). Patients indicated their age, gender, and whether they were a new or returning patient. Patients were asked to complete a two-page questionnaire designed to assess their resilience, sleep symptoms, current degree of subjective sleepiness, sleep quantity and quality, mental health symptoms, and other health symptoms. Upon completion of the study, the clinical investigators did not make any medical recommendations based on protocol design and IRB parameters.

### Measures

#### Pittsburgh sleep quality index (adapted): Sleep quantity and quality

Participants’ responses to five questions adapted from the validated Pittsburgh Sleep Quality Index (PSQI) (Johns, 1991). The five items used to assess sleep quantity and quality included: (1) “What time did you go to bed?”; (2) What time did you turn out the lights?”; (3) How many minutes until you fell asleep?”; (4) “How many minutes did you spend awake after falling asleep?”; (5) “What time did you finally wake in the morning?”. For consistency with other measures below, total minutes of sleep were multiplied by–1 so that higher values indicated lower sleep quantity. Participants also indicated their sleep quality on the previous evening by completing one question: “How did you sleep?” (1 = Very poorly to 5 = Very well). Similarly, responses were recoded such that higher values indicated worse sleep quality.

#### Epworth sleepiness scale: Daytime sleepiness

Degree of routine sleepiness was measured using the validated Epworth Sleepiness Scale (α = 0.77; 0 = no chance of dozing to 3 = high chance of dozing) (Buysse et al., 1989). As ESS represents a tool to report sleepiness symptoms over the last 2 weeks, additional questions were also asked to assess “*current sleepiness* and *current fatigue/tiredness*” using a standard, one-item measure developed by the sleep clinical investigators of the project (1 = Not at all to 10 = Very much so).

#### Brief resilience scale (modified): Resilience

Resilience was measured by asking participants to complete a modified version of the brief resilience scale (BRS), a reliable (α = 0.83), six-item measure (Smith et al., 2008). The six items used to assess resilience included: (1) “I tend to bounce back quickly after hard times”; (2) “I have a hard time making it through stressful events”; (3) “It does not take me long to recover from a stressful event”; (4) “It is hard for me to snap back when something bad happens”; (5) “I usually come through difficult times with little trouble”; (6) “I tend to take a long time to get over set-backs in my life”. For each item, participants used a slightly modified response scale (1 = Strongly disagree to 7 = Strongly agree). As suggested by Smith et al. (2008), items two, four, and six were reverse-coded, and the BRS score was calculated by taking the average of the six items.

#### Additional questions: Overall physical, mental health and adjunct sleep symptoms

- *Sleep symptoms* were also assessed using a standard, 19-item checklist developed by the sleep clinical investigators of the project (e.g., snored, pain interfering with sleep, nightmares, ground teeth during sleep, inability to move when going to or waking up from sleep). The checked items were summed to create a sleep symptoms score (ranging from 0–19), with higher scores indicating more sleep disruptive symptoms.
- *Physical and Mental Health Symptoms* were computed by summing the symptoms participants indicated that they experienced in the last month, using a checklist. Symptoms included difficulty with memory/concentration, changes in mood, changes in behavior, claustrophobia/anxiety, fatigue, weight gain, and “brain fog.” Scores could range from 0–7.
- *Overall review of symptoms and functioning* were estimated by summing the participant’s report of symptoms in the last month, including (1) headaches, (2) fever, (3) chills, (4) seasonal allergies, (5) nasal congestion, (6) cough, (7) runny nose, (8) night sweats, (9) hot flashes, (10) shortness of breath at rest, (11) shortness of breath with activity, (12) blood in urine, (13) blood in stools, (14) frequent urination, (15) dizziness, (16) diarrhea, (17) constipation, (18) stomach problems, (19) sour taste, (20) belching, (21) reflux, (22) swelling in feet, (23) swelling in legs, (24) leg cramps, (25) chest pain, (26) joint pain, (27) back pain, (28) rash, (29) excessive thirst, (30) dry mouth, (31) neck pain, (32) facial numbness, and (33) migraine. Scores could range from 0–33.

### Statistical analysis

Only participants with complete data (*n* = 190) were included in the analyses. Descriptive statistics were used to evaluate the average scores or frequencies across the variables included in the analyses. To examine associations between resilience and each of the sleep parameters (aim 1), Pearson correlations were computed. Any of the sleep parameters significantly associated with resilience were included in a multiple linear regression model. Additional models were computed to determine whether resilience was associated with each sleep parameter after accounting for demographic and health covariates. Finally, a set of models were conducted to test for a two-way interaction between resilience and mental health symptoms for each sleep parameter (aim 2). SPSS Version 20 was used for all analyses.

## Results

Participants had a mean age of 51 (*SD* = 15.57, range = 16–82), and 50% (*n* = 95) were women; see Table 1 for sample characteristics. The sample had an average modified BRS score of 4.67 (*SD* = 1.32, range = 1.17–7) reflecting a high resilience level. There was no significant association between age and BRS score (*r* = 0.08, *p* > 0.05). However, there was a significant gender difference in the BRS score, indicating that men (Mean = 5.04, *SD* = 1.14) had higher levels of reported resilience than women (Mean = 4.30, *SD* = 1.38, *t* (188) = 4.02, *p* < 0.001). Lastly, higher BRS scores were significantly associated with lower mental (*r* = −0.38, *p* < 0.01) and physical health (*r* = −0.16, *p* < 0.05) risk conditions.

### Resilience associated with sleep

**FIGURE 1:**
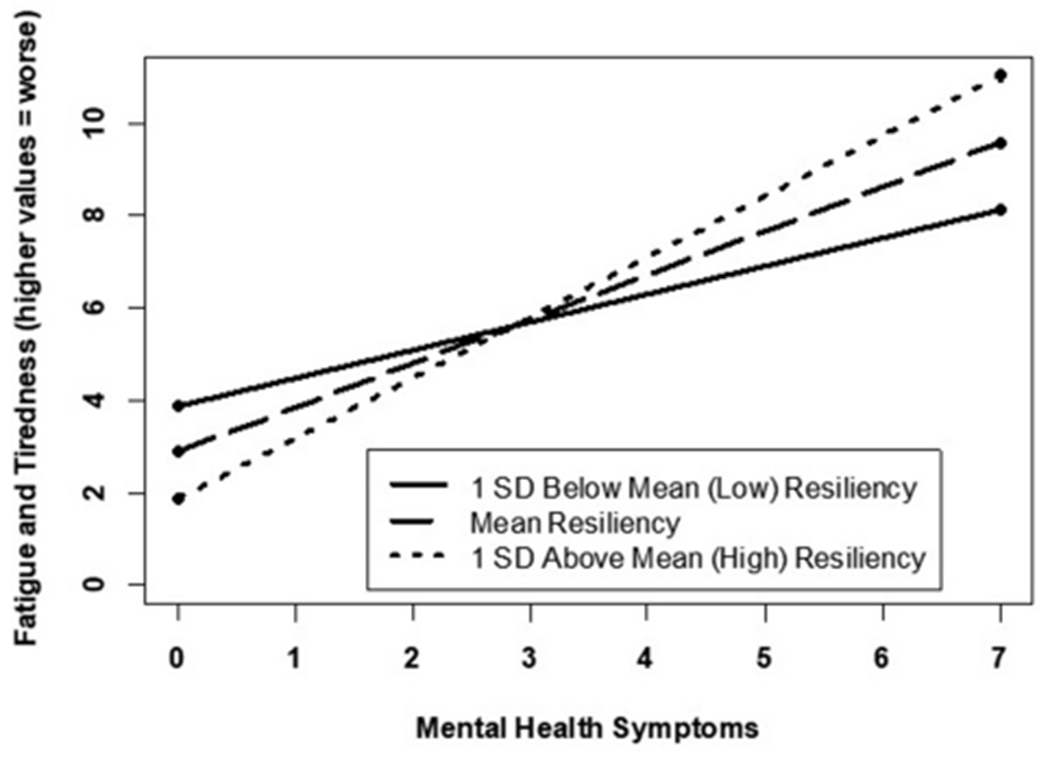
Mental health related to current fatigue/tiredness by varying resilience levels.

**FIGURE 2:**
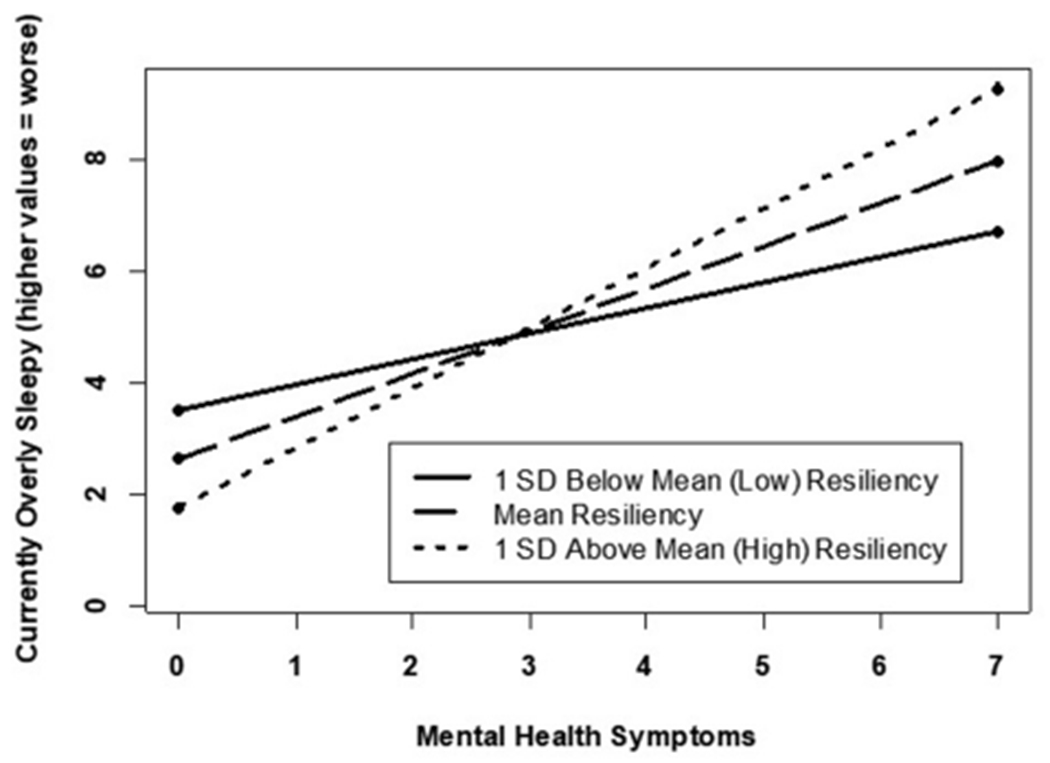
Mental health related to currently overly sleepy by varying resilience levels.

Correlational analyses (Table 2) revealed several associations between resilience and the sleep parameters. Specifically, lower levels of resilience were associated with shorter sleep duration, more disruptive sleep symptoms, greater sleepiness, reports of experiencing higher fatigue and tiredness, and feeling overly sleepy; these associations remained significant for symptoms of fatigue and tiredness even after adjusting for covariates (Table 3). Resilience was no longer associated with the other sleep parameters after adjusting for covariates. However, significant two-way interactions (BRS and mental health symptoms) were observed for (a) current fatigue and tiredness (Figure 1) and (b) currently overly sleepy (Figure 2). Although higher mental health symptoms were associated with greater current fatigue and tiredness as well as being overly sleepy, high resilience appeared to minimize this effect. Specifically, participants reporting between 1 and 3 mental health symptoms, high levels of resilience minimized the association between higher mental health symptoms and worse sleep quality symptoms. However, this minimizing effect was no longer evident in participants experiencing >3 mental health symptoms, who reported significantly higher symptoms of fatigue and sleepiness regardless of their resilience scores. Interestingly, this appears to demonstrate a threshold to the protective effect of resilience. After approximately three mental health symptoms, high resilience no longer appears to minimize the relationship between these symptoms and their manifestation in fatigue, tiredness, and sleepiness. These results support the pertinence of simultaneously evaluating potential risk and protective factors associated with poor mental and sleep health. Additionally, the results further support tailoring intervention programs or treatment plans depending upon an individual’s current mental health symptoms.

## Discussion

For the past several years, resilience has been investigated in the context of mental health research. Previous work has highlighted and enriched our understanding of the importance of resilience in human development (Cameron et al., 2006) and how it serves as an effective coping mechanism in the face of adversity across various psychosocial settings and events (e.g., personal and professional relationships, tragedies, etc.) (Fredrickson, 2001). Resilience has taken on growing importance in the context of a pandemic that will likely have both short term, intermediate and long term impacts on overall physical and mental health in addition to wellness. The amplified mental health crisis in the U.S. since the COVID-19 pandemic has economically burdened healthcare and the workforce through a rise in care for individuals with comorbid mental health conditions and chronic disease, as well as an estimated labor productivity loss of 17 billion dollars (Evernorth Health Inc, 2022). Therefore, encouraging strategies (i.e., behaviors, social engagement, or situational encounters) to promote resilience may represent a resourceful approach to improving health and wellness (Davydov et al., 2010) and even preventing and managing affective disorders like depression and anxiety (Connor and Zhang, 2006).

In this study, lower levels of resilience were associated with more sleep disturbances, while higher levels of resilience were associated with fewer sleep disturbances within our age and gender diverse participant sample (Pecillo, 2016). Additionally, higher levels of resilience were observed to lessen the negative impact of mental health symptoms on sleep quality, at least among those endorsing three or less mental health symptoms. In individuals experiencing more than three mental health symptoms, however, high resilience appeared to play little role in reducing the negative impact of mental health symptoms on sleep quality. It has been previously reported that poor sleep and mental health symptoms go hand-in-hand (Seelig et al., 2010). In particular, our study highlights that the relationship between sleep and/or resilience appears to be stronger for mental health than physical health. Likewise, prior literature has observed similar findings of resilience being associated with mental and physical health (Schure et al., 2013). Additionally, resilience was significantly associated with proxies of mental health, particularly depressive symptomology, even after adjusting for demographics, chronic pain, and other health measures. Our findings further suggest that, up to a point, higher levels of resilience may help to attenuate the relationship between mental health and sleep wellness. Given our observation of a potential threshold for the protective benefits of resilience on minimizing the association between sleep and mental health, our findings suggest that additional research is warranted to explore other protective factors (e.g., neighborhood cohesion, social support) that could further minimize the sleep-mental health association.

A focus on interventions to increase resilience begs the question of where resilience originates. The gene-environment interaction can play a role in determining an individual’s resilience (Davydov et al., 2010). There are identified genetic phenotypes relevant to resilience (e.g., reactivity), which may be amplified or weakened depending on specific environmental contexts (Davydov et al., 2010). For example, the number of positive emotions vs. negative emotions perceived during childhood can affect resilience (Ong et al., 2006). Interestingly, living in an environment that lacks any form of past adversity can also result in a reduced resilience (Davydov et al., 2010). Collectively, these factors can be viewed as sleep-independent factors, which can either enhance or reduce a person’s resilience. Based on the findings in the current study, patients presenting to a sleep center who have disrupted sleep, and potentially a sleep disorder and set of mental health problems, may be assisted by interventions to build their resilience (Connor and Zhang, 2006). For instance, cognitive behavioral strategies have proven beneficial for promoting resilience (Padesky and Mooney, 2012; Neenan, 2017). At a minimum, sleep providers could incorporate these resilience promoting behavioral techniques into the already established CBT-I approaches to manage patients who present concurrently with sleep concerns and suboptimal resilience.

This study also highlights the potential interactive role that resilience may play in the relationship between mental-health and sleep quality, making it a potentially important factor to consider in clinical treatment. In other words, incorporating methods to regularly evaluate resilience in patients with mental illnesses can be useful for predicting the potential manifestation and severity of sleep disturbance. The same strategy could also be adopted for those individuals initially presenting with sleep disruption, as a method of predicting potential functional impairment including affective symptoms. Awareness of the interaction between resilience and health might then be used as a proactive prevention and treatment strategy. This can be especially useful when designing strategies to mitigate the current and potentially impending negative mental health effects of the COVID-19 pandemic on those both within and served by the healthcare field (Evanoff et al., 2020; Gibson et al., 2020). For example, developing interventions to enhance resilience might help in the treatment of patients with newly emerging mental illnesses and/or sleep disruption.

While the results of this study are interesting, some limitations are worth mentioning. The first is the potential for selection bias. Here, the study was conducted using convenience sampling, and participant recruitment was limited to a clinical sleep population. Thus, results may disproportionately reflect the patterns of individuals experiencing and seeking to resolve sleep disorders. Additionally, the current study’s findings may not be generalizable to all patients experiencing poor sleep health. Future studies should build upon these findings by enrolling larger and more diverse patient samples. Moreover, this group represented a higher proportion of White participants than is representative of the general population. Also, although the BRS scale appears to be reliable and commonly used in the literature, it is based on individual experiences such as the perception of stressful events, stress recovery, and rumination on negative thoughts. This means that the scale is potentially subject to self-reporting biases. Additionally, the BRS scale does not measure potential sources of resilience such as social cohesion and spirituality (Fredrickson and Joiner, 2002). We also acknowledge that all analytic models were run using cross-sectional data, which limits the ability to state whether levels of resilience were driving the minimizing effect of mental health symptoms on sleep quality. Lastly, this study did not include a common standardized scale of mental health symptoms, such as a depressive symptomology scale or positive/negative mood scale. The study also did not include information on the clinical background (medical diagnoses and treatments) of the patients included in the study. It is conceivable that including such information could influence the current study’s conclusions. Despite these limitations, this study highlights a relationship between resilience, physical and mental health symptoms, and sleep disturbances that has been under-reported in the literature—and potentially useful for future research and clinical treatment.

In summary, sleep and mental health are often intertwined. Strikingly, our study investigators observed in the U.S. an approximate proportional increase of 333% COVID-19 cases (23 million in 2021 vs. 100 million in 2022) and 186% COVID-19 deaths (385 thousand in 2021 vs. 1.1 million in 2022) relevant to the COVID-19 pandemic from January 2021 and December 2022 using the JHU COVID-19 dashboard (Evernorth Health Inc, 2022). This stark increase in COVID cases and deaths highlights the relevance of continued evaluation of sleep wellness and mental health. Additionally, it also signifies the pertinence of investigating how protective factors, such as resilience, can minimize the magnified U.S. population trend toward poor mental and sleep health. Moreover, resilience may serve as an additional tool for developing personalized and targeted approaches to clinical care for either or both.

## Figures and Tables

**TABLE 1:** Sample characteristics (*n* = 190).

	*n* (%)	Range	Mean	SD
Age, years	-	16-82	51.16	15.57
Gender, female	95 (50.0)	-	-	-
Mental health symptoms	-	0–6	1.87	1.39
Other health conditions	-	0–21	4.24	3.54
Sleep quantity	-	3–12.25	7.17	1.57
Sleep quality, last night	-	1–5	3.02	1.06
Sleep symptoms	-	1–13	4.46	2.49
Epworth sleepiness scale	-	8–24	14.65	3.90
Current fatigue and tiredness	-	1–10	5.60	2.84
Current overly sleepy	-	1–10	4.36	2.77
Mean modified BRS	-	1.17–7	4.67	1.32

BRS represents the brief resilience scale.

**TABLE 2:** Resilience as it relates to each sleep parameter.

	Mean BRS
Sleep quantity	−0.17*
Sleep quality, last night	0.14
Sleep symptoms	−0.22**
Epworth sleepiness scale	−0.15*
Current fatigue and tiredness	−0.34**
Current overly sleepy	−0.29**

* *p* < 0.05.

** *p* < 0.01.

BRS represents the brief resilience scale.

**TABLE 3:** Resilience as it relates to each sleep parameter after adjusting for covariates.

	Sleep quantity	Sleep symptoms	Epworth sleepiness scale	Current fatigue and tiredness	Current overly sleepy
Age	−0.13 (0.01)	0.00 (0.01)	−0.21** **(0.02)**	−0.02 (0.01)	−0.06 (0.11)
Gender	0.07 (0.24)	−0.10 (0.30)	−0.01 (0.57)	0.06 (0.36)	0.32 (0.37)
Mental health symptoms	0.12 (0.10)	0.34*** **(0.12)**	0.10 (0.24)	0.42** **(0.15)**	0.34*** **(0.15)**
Other health conditions	−0.06 (0.04)	0.41*** **(0.05)**	0.17* **(0.09)**	0.16* **(0.05)**	0.16* **(0.06)**
Mean BRS	−0.11 (0.10)	−0.05 (0.12)	−0.07 (0.23)	−0.13* **(0.14)**	−0.12 (0.15)

Numbers included in table represent standardized beta coefficients and (standard errors).

Bold font represents significant associations.

* *p* < 0.05.

** *p* < 0.01.

*** *p* < 0.001.

BRS represents the brief resilience scale.

## Data Availability

The raw data supporting the conclusions of this article will be made available by the authors, without undue reservation.
